# Right upper lobectomy with mediastinal dissection under uniportal video-assisted thoracoscopic surgery for lung cancer in a patient with a right-sided aortic arch: a case report

**DOI:** 10.1186/s13019-024-02627-9

**Published:** 2024-03-19

**Authors:** Hidenori Goto, Kozo Nakanishi

**Affiliations:** Department of General Thoracic Surgery, National Hospital Organization, Saitama Hospital, Suwa 2-1, Wako, Saitama 351-0102 Japan

**Keywords:** Right-sided aortic arch, Uniportal VATS, Lung cancer, Mediastinal lymph node dissection

## Abstract

**Background:**

A right-sided aortic arch is a rare congenital vascular structure variation. Right lobectomy is not commonly performed on patients with such a condition. Further, there are no reports on lobectomy under uniportal video-assisted thoracoscopic surgery (VATS) in this patient group.

**Case presentation:**

A 67-year-old man with a right-sided aortic arch and Kommerell diverticulum underwent right upper lobectomy with mediastinal lymph node dissection under uniportal VATS for primary lung cancer. Due to the right descending aorta, which narrows the space of the dorsal hilum, handling of the stapler for stapling the right upper lobe bronchus from the uniport in the 6th intercostal space at the medial axillary line can be challenging. This issue was resolved by manipulating the staple over the azygos vein toward the inferior margin of the aortic arch. Via mediastinal lymphadenectomy, we found that the right recurrent laryngeal nerve branched from the right vagus nerve and hooked around the right-sided aortic arch.

**Conclusions:**

Right lobectomy with mediastinal lymph node dissection under uniportal VATS can be performed for lung cancer in patients with a right-sided aortic arch.

## Background

A right-sided aortic arch is a rare congenital anomaly, with retention of the 4th branchial arch artery on the right side. The incidence of this condition is approximately 0.1% [1]. In the past 15 years, only eight cases of a right-sided aortic arch have been reported among patients who had undergone lobectomy for right lung cancer [2]. To the best of our knowledge, this is the first case report in which right upper lobectomy with mediastinal lymph node dissection (ND2a-1) was performed under uniportal video-assisted thoracoscopic surgery (VATS) in a patient with right lung cancer. Herein, we report a case of lung cancer with a right-sided aortic arch.

## Case presentation

**Fig. 1:**
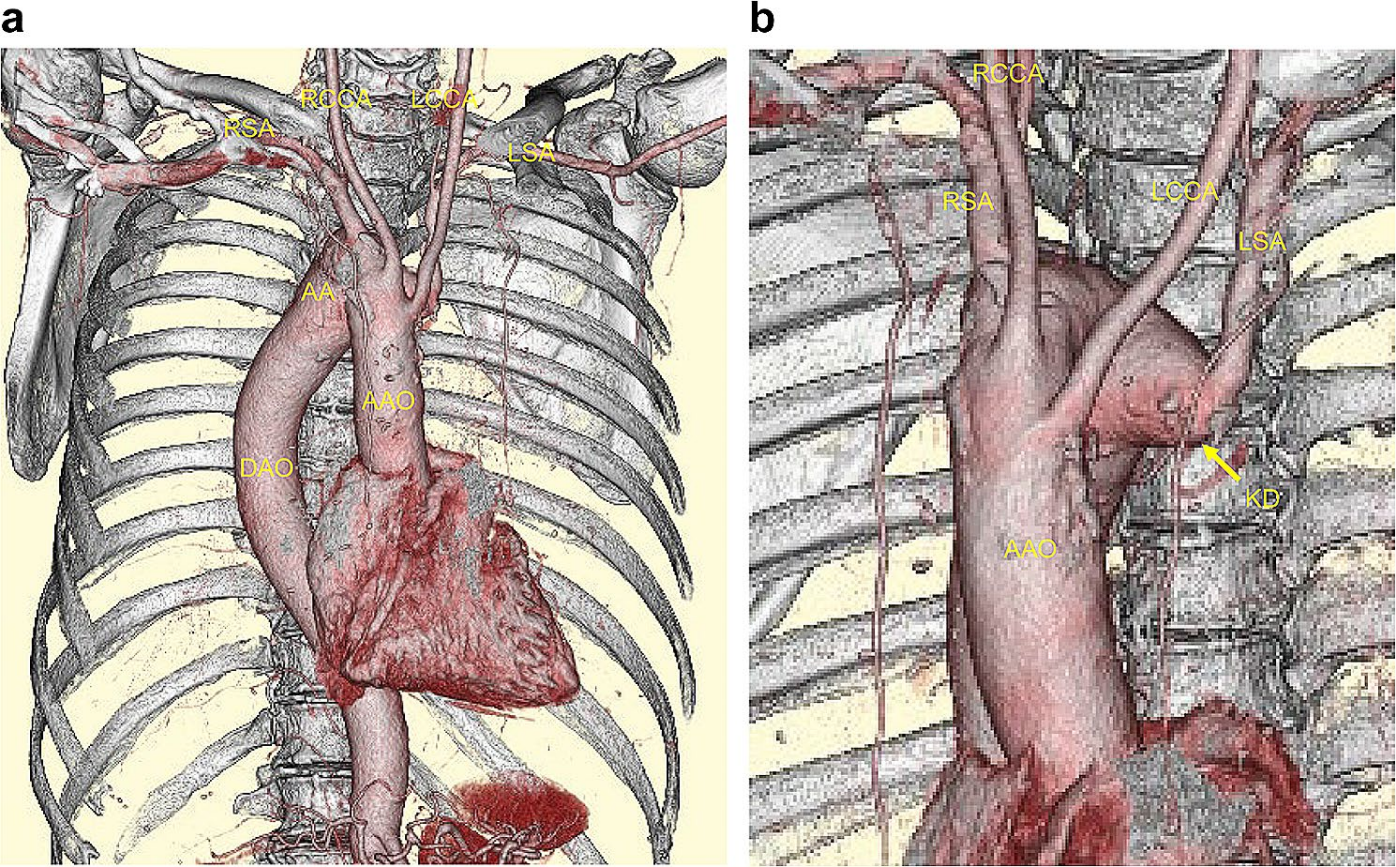
Three-dimensional computed tomography scan image of the right aortic arch (**a**) The anterior view shows the right aortic arch and the right descending aorta running on the right side of the vertebral body (**b**) The lateral view shows the so-called Kommerell diverticulum of the left subclavian artery AAO, ascending aorta; DAO, descending aorta; LCCA, left common carotid artery; LSA, left subclavian artery; RCCA, right common carotid artery; RSA, right subclavian artery; KD, Kommerell diverticulum

**Fig. 2:**
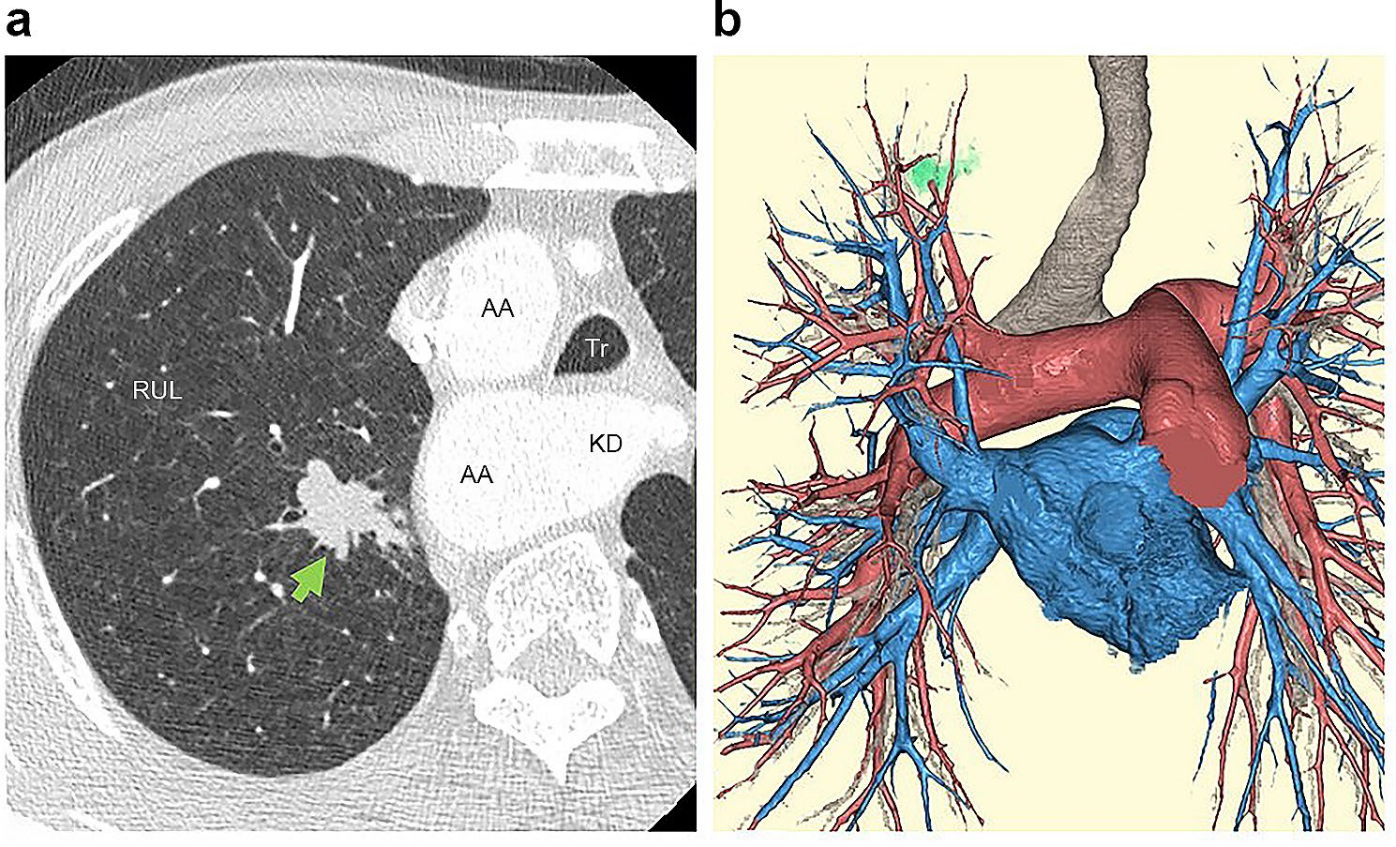
Preoperative computed tomography (CT) scan (**a**) The CT scan image revealed a lung nodule in the right S1 (green arrow) (**b**) The branching of the pulmonary artery, vein, and bronchi were normal AA, aortic arch; KD, Kommerell diverticulum; RUL, right upper lobe; Tr, trachea

**Fig. 3:**
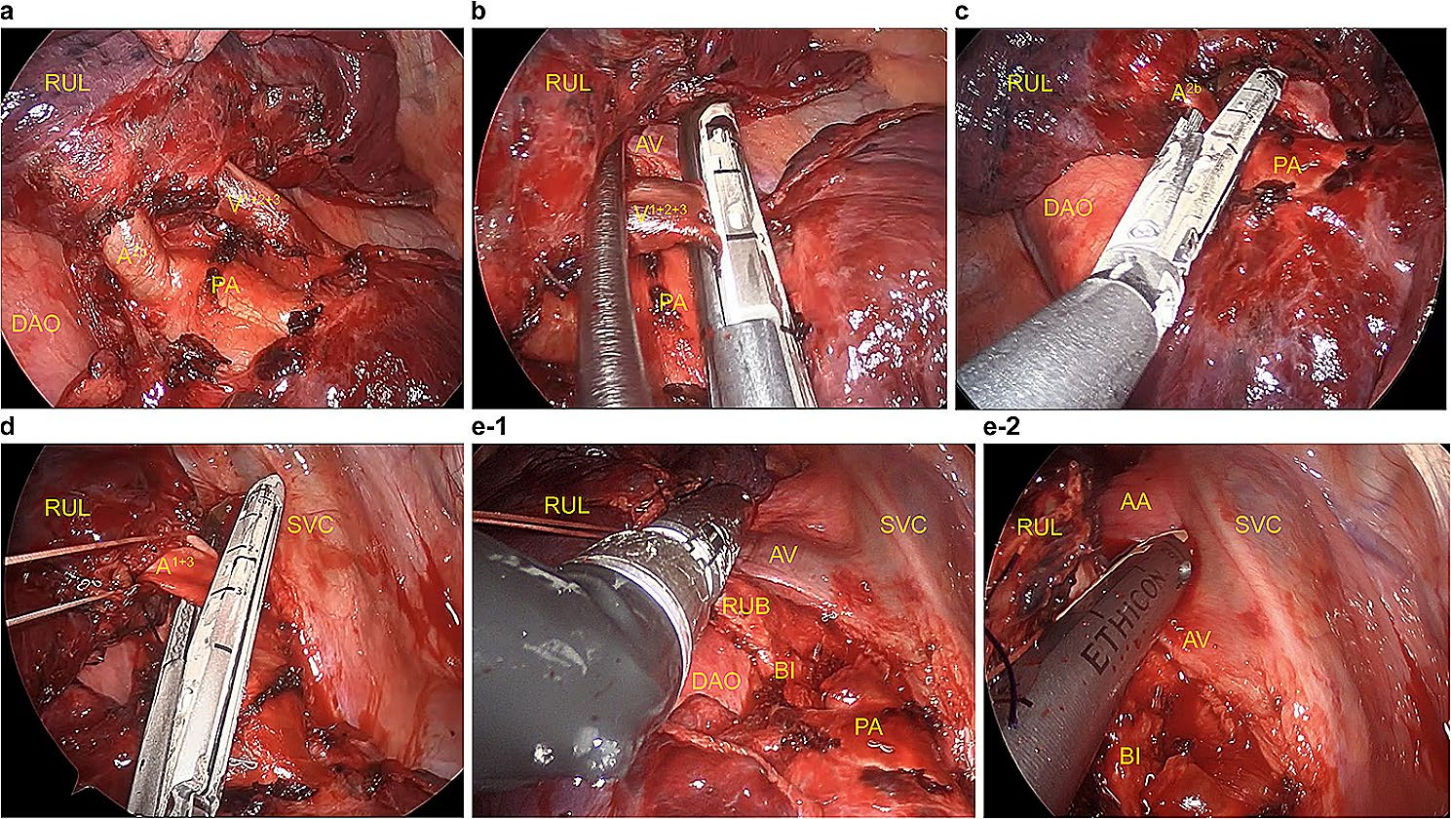
(**a**) Operative findings of the right upper lobectomy Intraoperative findings after dividing the interlobar fissure. (**b**) The right upper lobe pulmonary vein (V1 + 2 + 3). (**c**) The truncus anterior artery (A1 + 3). (**d**) The ascending artery (A2b) was cut using staplers Endo-GIA Ultra Universal Staples; Medtronic plc., Dubin, Ireland) (**e-1**) The stapler was advanced over the descending aorta, thereby causing malpositioning of the planned resection line of the right upper lobe bronchus (**e-2**) By manipulating the staple toward the inferior margin of the aortic arch, the right upper lobe bronchi were dissected appropriately PA, pulmonary artery; AA, aortic arch; AV, azygos vein; RUB, right upper lobe bronchus; RUL, right upper lobe; BI, bronchus intermedius; DAO, descending aorta; SVC, superior vena cava

**Fig. 4:**
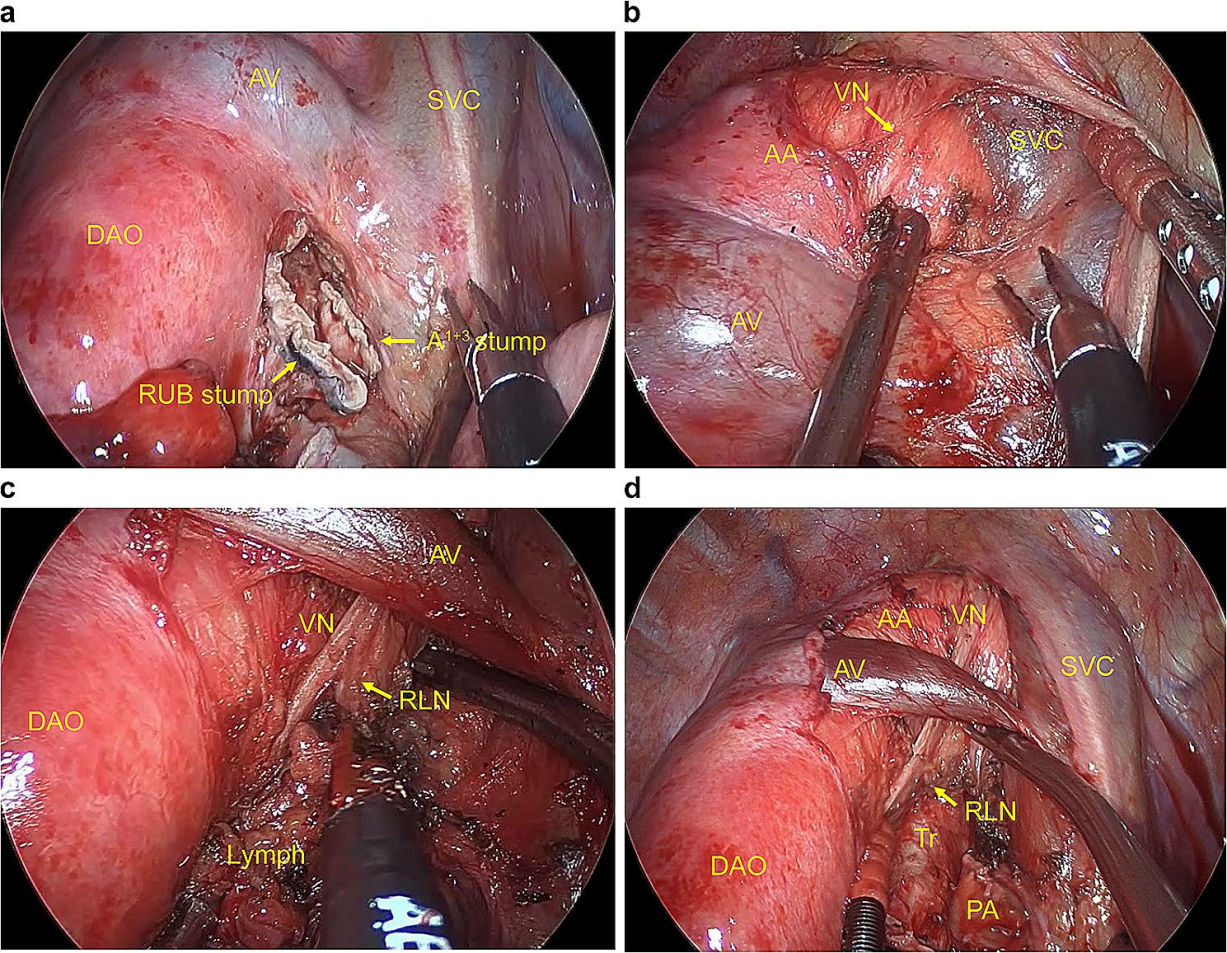
Operative findings of the right upper mediastinum (**a**) An intraoperative view before the upper mediastinal lymph node dissection (**b**) The right vagus nerve was identified at the cranial side of the azygos vein while dissecting the mediastinal lymph node from SVC. (**c**) The recurrent laryngeal nerve, which branched from the vagus nerve, was identified at the caudal side of the azygos vein while dissecting the mediastinal lymph node from the AA. (**d**) An intraoperative view after the upper mediastinal lymph node dissection PA, pulmonary artery; RLN, recurrent laryngeal nerve; VN, vagus nerve; Tr, trachea; AA, aortic arch; AV, azygos vein; RUB, right upper lobe bronchus; DAO, descending aorta; SVC, superior vena cava

A 67-year-old man was monitored for a right-sided aortic arch and Kommerell diverticulum (Fig. 1a and b). There was no significant medical history other than the right-sided aortic arch. He had a smoking history of at least 50 pack-years and currently smoke. The patient was symptom-free, and there were no remarkable findings on physical examination. The tumor markers carcinoembryonic antigen, CYFRA, and pro gastrin releasing peptide and spirometry were within normal limits. Echocardiography revealed no cardiac abnormalities. The most recent chest computed tomography (CT) scan revealed a 20-mm × 18-mm solid nodule in the right S1 (Fig. 2a). The nodule was suspected to be malignant based on the increase in lesions over time on CT scans. A systemic CT, brain magnetic resonance imaging (MRI), and bone scintigraphy examination revealed no other lesions. There were no anatomic variations in the pulmonary vessels or bronchi (Fig. 2b). Right upper lobectomy with ND2a-1 under uniportal VATS was performed as stage IA2 right upper lobe lung cancer was suspected. Using a soft tissue retractor, a 4-cm incision in the 6th intercostal space at the medial axillary line was made as the main manipulation port. The operative findings confirmed the presence of a right descending aorta that runs on the right ventral side of the vertebral body and that narrows the space of the dorsal hilum. After dividing the interlobar fissure with a stapler (Endo-GIA Ultra Universal Staples; Medtronic plc., Dubin, Ireland), the V^1 + 2+3^, A^2b^, and A^1 + 3^ were detached and divided sequentially (Fig. 3a–d). After dissecting the right upper lobe bronchus, due to the right descending aorta, handling of a stapler (Powered Echelon Flex GST System 45; ETHICON Endo-Surgery, inc., Cincinnati, OH, USA) used to staple the right upper lobe bronchus located on the dorsal hilum was challenging (Fig. 3e-1). This issue was resolved by manipulating the staple over the azygos vein toward the inferior margin of the aortic arch (Fig. 3e-2). The diagnosis of the intraoperative frozen section was carcinoma. During lymph node dissection of the upper mediastinum, the right vagus nerve was identified at the cranial side of the azygos vein (Fig. 4b). Next, dissection was performed in the caudal direction. The right recurrent laryngeal nerve was found to branch from the right vagus nerve just beneath the right-sided aortic arch (Fig. 4c), and the mediastinal lymph node was subsequently dissected (Fig. 4d). The operative time was 325 min, and the volume of blood loss was 59 mL. The patient was discharged on postoperative day 8 without any complications such as hoarseness. The pathological diagnosis was small cell lung cancer. The tumor had a diameter of 21 mm × 14 mm with histopathological features of pm0, pl0, ly0, and v1. Four #2R+#4R lymph nodes, one #11 lymph node, and six #12u lymph nodes were retrieved, but there was no #10R lymph node. No lymph node metastasis was detected. After 8 months of adjuvant chemotherapy for the stage IA3 tumor, no recurrence of lung cancer was observed by positron emission tomography-CT and brain MRI.

## Discussion and conclusions

Given how uncommon the present case is, it is crucial to determine the anatomical features of the right intrathoracic space in patients who had undergone for primary lung cancer and had a right-sided aortic arch. According to the classification of Edwards et al. [3], our case was type IIIB, which is characterized by the dilated stem of the left subclavian artery. This condition is referred to as a Kommerell diverticulum, which runs from the right to left side behind the esophagus. As a result, the right descending aorta after branching a Kommerell diverticulum runs more ventral to the vertebral body than the left descending aorta in a normal anatomy. Therefore, if the dorsal hilum is pushed more ventrally, the right upper lobe bronchus located on the dorsal hilum is more challenging to dissect.

Uniportal VATS lobectomy is an effective surgical procedure [4]. The greatest strength of the technique is its prospective benefits for patients, including reduced postoperative pain in comparison to other multiportal approaches, speedy patient recovery, short postoperative hospital stay duration, and improved clinical cosmetic outcomes [5]. However, compared with multiportal VATS, it can be more challenging to interfere with thoracoscopic instruments used to expand the operative field. The direction of insertion is essential for the stapler that is deployed in a fan shape from the uniport because it is necessary to secure a space behind the separation. During resection of the right upper lobe bronchus located on the dorsal hilum, the presence of the right descending aorta narrowing the space of the dorsal hilum prevented the stapler from advancing cranially from the uniport. Therefore, this issue was resolved by devising the orientation of the stapler.

Regarding lymph node dissection, multiple VATS has advantages in terms of the amount of the dissected lymph nodes [6]. The direction of insertion from the uniport may limit the operative field of view for the surgeon. The reported uniport VATS lobectomy often uses the anterior approach [5], but the direction of insertion from the uniport may limit the operative field of the dorsal view of the hilum and superior vena cava. In right upper lobectomy and ND2a-1, the main manipulation port was made in the 6th intercostal space at the medial axillary line because this site can conveniently show the dorsal view of the hilum and superior vena cava, which is beneficial to lobectomy and lymph node dissection. In the present case, the direction of the insertion from the uniport might have allowed an easier dissection of the upper lobe bronchus located on the dorsal hilum and the dissection of the lymph nodes dorsal to superior vena cava.

The aortic arch is overhanging the trachea. Hence, dissection of the upper mediastinal lymph nodes in patients with a right-sided aortic arch differs from the routine dissection. Matsubara et al. hypothesized that the left and right upper mediastinal lymph nodes are separated by the aortic arch, not the trachea [7]. In addition, the left–right asymmetry of the upper mediastinal lymph nodes is caused by the retention and regression of the left and right branchial arch artery. According to this hypothesis, the upper mediastinal lymph nodes are classified as periaortic lymph nodes on the side where the branchial arch artery retentions are located [8]. Dissection of the upper mediastinum of the right aortic arch should be performed according to left upper mediastinum dissection. In a previous report, dissection of the upper mediastinum of the right aortic arch was performed according to left upper mediastinum dissection based on anatomical characteristics [2]. Regarding the differences between the left upper mediastinal dissection in a normal anatomy and the right upper mediastinal dissection in cases of a right-sided aortic arch, the azygos vein and superior vena cave are observed and the right recurrent laryngeal nerve must be identified without the ductus arteriosus. Thus, in the present case, the dissection areas are to the right lateral side of the aortic arch between the upper and lower edges of the aortic arch and to the right lateral edge of the trachea between the lower edge of the aortic arch and the head of the right main pulmonary artery. The ventral side of the dissection border becomes the superior vena cava.

To the best of our knowledge, this is the first detailed report on right upper lobectomy with ND2a-1 under uniportal VATS for primary lung cancer in a patient with a right-sided aortic arch and Kommerell diverticulum. The surgical procedure was performed despite the potential risks caused by anatomical changes.

## Data Availability

All data generated or analyzed during this study are included in this published article.

## Abbreviations

VATS: Video-assisted thoracoscopic surgery
CT: Computed tomography
